# Late antiretroviral therapy initiation and associated factors among children on antiretroviral therapy at University of Gondar Comprehensive Specialized Hospital, Gondar, Northwest Ethiopia: a cross-sectional study

**DOI:** 10.1186/s13104-019-4279-z

**Published:** 2019-05-07

**Authors:** Getaneh Mulualem Belay, Eshetu Haileselassie Engeda, Amare Demsie Ayele

**Affiliations:** Department of Pediatrics and Child Health Nursing, School of Nursing, College of Medicine and Health Sciences, University of Gondar, Gondar, Ethiopia

**Keywords:** Children, Ethiopia, Late ART initiation, University of Gondar Comprehensive Specialized Hospital

## Abstract

**Objective:**

Highly active antiretroviral therapy reduces HIV related morbidity and mortality dramatically. Despite this fact, late ART initiation poses poor treatment outcome in pediatrics. However, the information is scarce in Ethiopia. Therefore, the study was aimed at determining the burden of late ART initiation and its associated factors among children on ART. Cross-sectional study was conducted among 422 children selected by simple random sampling. Patient charts were reviewed using pretested and structured data abstraction tool. Binary logistic regression model was fitted.

**Results:**

A total of 402 child records with a completeness rate of 95.3% were included. The overall proportion of late antiretroviral therapy initiation among children on antiretroviral therapy was 53.2% (95% CI 48.5–58.4%). Under-5 years of age [AOR: 2.165 (95% CI 1.341, 3.495)], rural residence [AOR: 1.825 (95% CI 1.052, 3.166)], taking non-ART medication [AOR: 2.237 (95% CI 1.212, 4.130)], past opportunistic infection [AOR: 2.548 (95% CI 1.554, 4.178)], unmarried caregiver [AOR: 1.618 (95% CI 1.023, 2.559)], male caregiver [AOR: 1.903 (95% CI 1.026–3.527)] and null ANC visit [AOR: 1.721 (95% CI 1.077, 2.752)] were significantly associated factors. There is high burden of late ART initiation in children. Thus, focus should be started from pregnancy.

## Introduction

Highly active antiretroviral therapy (HAART) is a combination of three or more antiretroviral drugs that substantially minimize HIV related morbidity and mortality [1]. However, late antiretroviral therapy (ART) initiation remains a major problem in the world, especially in low income countries [2]. It increases child morbidity, hospitalization and mortality [3]. Most children on ART faced treatment failure due to initiating ART at low CD4 count [4]. Nowadays, children on treatment encountered many challenges as in CD4 cell depletion, impaired CD4 recovery, increased mortality within a year of initiation, decreased life expectancy, and increased death at home before medical advice or ART initiation. These were due to high rates of late ART initiation [5, 6].

Despite progressive improvement in ART coverage, there is still high burden of late ART initiation in developing countries like 54.4% in Uganda [7, 8], 68.2% in South African [9], 48% in Mozambique [10] and 58.3% in Addis Ababa, Ethiopia [11].

Different strategies were employed to overcome it as in World Health Organization (WHO) frequently updated ART eligibility criteria [12]; but it remains high in developing countries [13]. In addition, shorter waiting time, supportive counseling and free charge prophylaxis were implemented. Nonetheless, it has not significantly reduced yet. Moreover, it contravenes the 2020 Ethiopia’s 90% ART coverage plan.

Late HIV diagnosis [14], being maternal orphan [15, 16], parental lower education and unemployment have highly determined late ART initiation [7] in children. Though, socio-demographic characteristics were clearly described in different literatures, clinical characteristics like HIV status of parents, year of initiation, relation to children and other treatment related characteristics were not clearly stated.

Furthermore, information on associated factors is scarce in the study area. Therefore, the study aimed to determine late ART initiation and its associated factors among children on ART. The study helps higher officials, managers and health policy makers to take appropriate measures. Furthermore, the findings of this study will serve as baseline for future researches.

## Main text

### Methods and materials

#### Study design, period and setting

An institution-based cross-sectional study was conducted from March, 28/2017 to April, 28/2017 at University of Gondar Comprehensive Specialized Hospital (UoGCSH) ART clinic.

#### Population and selection criteria

All HIV positive children **<** 15 years who had been enrolled in HAART at UoGCSH ART clinic from January 1st 2007 to December 30th 2016 were the study population. Complete child records available at patient database were included in the study.

#### Sampling technique and procedures

Totally, 617 children were enrolled in ART from January 1st 2007 to December 30th 2016. About 422 child records were taken by simple random sampling using computer generated random numbers. Finally, 402 charts were fulfilled the inclusion criteria and analyzed in the study.

#### Data collection tools and procedures

Data were collected by three ART expert nurses using data extraction tool.

#### Operational definitions

Late ART initiation (*YES/NO*): initiation of ART at CD4 count or percent of **< **250 cells/mm^3^ for children ≥ 5 years; **< **15% (350 cells/mm^3^) total lymphocyte count for children 3–5 years; below 20% (750 cells/mm^3^) for 12–35 months children and below 25% (1500 cells/mm^3^) for children 11 months and below [7, 17]; Or initiate ART at WHO stage 3 or 4 [6, 10].

#### Data quality control

One day training was given for data collectors. Pretest was done at UoGCSH on 22 child records. Data retrieval was monitored and checked daily by investigators. Data were entered using EPI Info-7 which reduces data entry errors.

#### Data processing and analysis

We used SPSS-20 for data analysis. Standard deviations, means, medians and inter-quartile ranges were used for data summarization. Texts and tables were used for data presentation. Variables with P-value < 0.2 were entered into multivariable analysis. Logistic regression model was fitted. Finally, variables with P-value < 0. 05 were considered statistically significant with late ART initiation.

### Results

#### Socio-demographic characteristics of children on ART

A total of 402 child records with completeness rate of 95.3% were included in the study. The mean age of children at ART initiation was 74.2 months in that 17 (4.2%) were < 11 months, 73 (18.2%) were from 12 to 35 months, 80 (19.9%) were from 36 to 60 months and 232 (57.7%) were above 5 years. From all children on ART, 208 (51.7%) were females by sex, 308 (76.6%) were urban by residence and 327 (81.3%) were living with their parents.

#### Baseline socio-demographic characteristics of caregivers’ of children on ART

From all caregivers, 81.8% were females, 84.8% were under 40 years, 70.9% of them were mothers, 62.2% of both parents are alive, about 84.1% were unemployed, and 50.7% were married; 52% and 51.1% of caregiver’s HIV status were unknown and HIV positive on ART respectively (Table 1).

**Table 1:** Baseline socio-demographic characteristics of caregivers for children on ART at University of Gondar Comprehensive Specialized Hospital, 2017 (n = 402)

Variables	Frequency (n)	Percentage (%)
Age		
< 40 years	341	84.8
≥ 40 years	61	15.2
Sex		
Male	73	18.2
Female	329	81.8
Marital status		
Married	204	50.7
Unmarried	198	49.3
Occupation		
Employed	64	15.9
Unemployed	338	84.1
Relation to child		
Mother	285	70.9
Father	41	10.2
Other relative	76	18.9
Antenatal history		
No	133	46.7
Yes	152	53.3
PMTCT		
No	123	77.4
Yes	36	22.6
Parental survival status		
Mother alive only	77	19.1
Father alive only	30	7.5
Both alive	250	62.2
Neither alive	45	11.2
HIV status of caregiver		
Positive	174	43.3
Negative	19	4.7
Unknown	209	52
Caregiver on ART		
No	85	48.9
Yes	89	51.1

n = sample size included in the analysis

#### Baseline clinical and treatment related characteristics of children on ART

From all children on ART, 72.4% were stayed below 6 months from test to enrollment to care, 74.4% had no other medication than ART, 53.2% had no past opportunistic infection before/at enrollment to care, 83.6% had no any TB treatment history, 80.6% had no any chronic diseases, 33.8% had WHO stage I during HIV test confirmed; whereas 42.7% had WHO stage III and IV at the time of ART initiation and 79.8% were initiated ART with CD4 count > 250 cells/mm^3^; whereas 50.7% were initiated with CD4 percent > 25% (Table 2).

**Table 2:** Baseline clinical and treatment related characteristics of children on ART at University of Gondar Comprehensive Specialized Hospital, 2017 (n = 402)

Variables	Frequency (n)	Percentage (%)
Duration between HIV test confirmed and enrollment		
< 6 months	291	72.4
6–12 months	46	11.4
> 12 months	65	16.2
Any medication before ART initiation		
No	299	74.4
Yes	103	25.6
Past OI at enrollment		
No	214	53.2
Yes	188	46.8
Cotrimoxazole prophylaxis		
No	42	10.4
Yes	360	89.6
INH prophylaxis		
No	338	84.1
Yes	64	15.9
TB treatment history		
No	336	83.6
Yes	66	16.4
Chronic disease		
No	324	80.6
Yes	78	19.4
Immunization status		
Appropriate for age	323	80.3
Not appropriate for age	67	16.7
Not Immunized	12	3
WHO stage at HIV test		
I	136	33.8
II	133	33.1
III	98	24.4
IV	35	8.7
Functional status (≥ 5 years)		
Working	120	47.8
Ambulatory/bedridden	131	52.2
Developmental milestone (< 5 years)		
Appropriate ranges for age	116	76.8
Developmental delay/regression	35	23.2
CD4 count (> 5 years)		
< 250 cells/mm^3^	50	20.2
≥ 250 cells/mm^3^	198	79.8
CD4 percentage (≤ 5 years)		
< 15%	37	24.0
15–25%	39	25.3
> 25%	78	50.7
WHO stage at ART initiation		
I	94	23.4
II	136	33.8
III	130	32.3
IV	42	10.5
Year of ART initiation		
2007–2008	69	17.2
2009–2011	137	34.1
2012–2014	115	28.6
2015–2016	81	20.1

#### Late ART initiation among children on ART

The overall proportion of late ART initiation among 402 children on ART was 53.2% (95% CI 48.5–58.4%). Of these, 47.2% were by WHO stage III and IV only, 71 (33.2%) were by both WHO staging and CD4 count/percentage, and 42 (19.6%) were by CD4 count/percent only. The median CD4 count and percentage at initiation of ART were 415.5 cells/mm^3^ (IQR: 363–446) and 26% (IQR: 23–27) respectively. Over the years, the proportion of late ART initiation decreases inconsistently from 53.6% (2007–2009) to 44.4% (2015–2016).

#### Factors associated with late ART initiation among children on ART

In the multivariable analysis, age of children, child residence, any non-ART medication, past opportunistic infection, antenatal history, sex and marital status of caregiver were significantly associated with late ART initiation.

The odds of being late initiated for ART among children < 5 years were 2 times more likely [AOR: 2.165 (95% CI 1.341–3.495)] as compared to those > 5 years of age.

The odds of being late ART initiated among rural children were almost two times more likely [AOR: 1.825 (95% CI 1.052, 3.166)] as compared to urban residents.

The odds of late ART initiation among children experienced past opportunistic infection before ART initiation were 2.5 times more likely [AOR: 2.548 (95% CI 1.554–4.178)] as compared to those who had not.

Besides, the odds of being late ART initiated among children who took medication before ART initiation was 2 times more likely [AOR: 2.237 (95% CI 1.212–4.130)] as compared to those who had not taken.

Additionally, the odds of late ART initiation among children from male caregivers were nearly 2 times more likely [AOR: 1.903 (95% CI 1.026–3.527)] as compared to children from female caregivers.

Moreover, the odds of late ART initiation among children from unmarried caregivers were 1.6 times more likely [AOR: 1.618 (95% CI 1.023–2.559)] as compared to children from married caregivers.

Finally, children from mothers who had no ANC follow up increases the odds of late ART initiation by 1.7 times [AOR: 1.721 (95% CI 1.077–2.752)] as compared to children from mothers who attended ANC (Table 3).

**Table 3:** Bivariable and multivariable logistic regression analysis of factors associated with late ART initiation among children on ART at University of Gondar Comprehensive Specialized Hospital, Northwest Ethiopia, 2017 (n = 402)

Variables	ART late initiated		COR, (95% CI)	AOR, (95% CI)
	Yes	No		
Age				
≤ 60 months	101	69	1.541 (1.033–2.300)	*2.165 (1.341*–*3.495)*
> 60 months	113	119	1	1
Sex				
Male	112	82	1.419 (0.957–2.104)	1.399 (0.888–2.204)
Female	102	106	1	1
Residence				
Urban	150	158	1	1
Rural	64	30	2.247 (1.380–3.660)	*1.825 (1.052*–*3.166)*
Child living condition				
With parent	171	156	1	1
Without parent	43	32	1.226 (0.739–2.034)	0.751 (0.366–1.543)
Duration between HIV test confirmed and enrollment				
< 6 months	158	133	1	1
6–12 month	17	29	0.493 (0.260–0.937)	0.499 (0.241–1.034)
> 12 months	39	26	1.263 (0.731–2.182)	1.176 (0.621–2.227)
Any medication before ART initiation				
No	139	160	1	1
Yes	75	28	3.083 (1.889–5.033)	*2.237 (1.212*–*4.130)*
Past OI				
No	83	131	1	1
Yes	131	57	3.627 (2.395–5.494)	*2.548 (1.554*–*4.178)*
TB treatment history				
No	161	175	1	1
Yes	53	13	4.431 (2.329–8.432)	1.925 (0.932–3.974)
Chronic disease				
No	157	167	1	1
Yes	57	21	2.887 (1.673–4.983)	0.958 (0.413–2.224)
Caregiver age				
< 40 years	179	162	1	1
≥ 40 years	35	26	1.218 (0.703–2.112)	0.827 (0.403–1.696)
Caregiver sex				
Male	46	27	1.633 (0.969–2.752)	*1.903 (1.026*–*3.527)*
Female	168	161	1	1
Marital status				
Unmarried	117	81	1.593 (1.074–2.364)	*1.618 (1.023*–*2.559)*
Married	97	107	1	1
Occupation				
Unemployed	185	153	1.459 (0.853–2.496)	1.211 (0.639–2.296)
Employed	29	35	1	1
Relation to child				
Mother	146	139	1	1
Father	26	15	1.650 (0.839–3.246)	1.086 (0.290–4.068)
Other relative	42	34	1.176 (0.707–1.955)	0.983 (0.325–2.980)
Maternal antenatal history				
No	80	53	2.075 (1.369–3.106)	*1.721 (1.077*–*2.752)*
Yes	64	88	1	1
Parental survival status				
Mother alive only	35	42	0.745 (0.446–1.244)	0.499 (0.230–1.083)
Father alive only	21	9	2.086 (0.919–4.733)	1.047 (0.325–3.367)
Neither alive	26	19	1.223 (0.644–2.324)	1.145 (0.419–3.127)
Both alive	132	118	1	1
Caregiver HIV status				
Positive	92	82	1	1
Negative	9	10	0.802 (0.311–2.071)	0.361 (0.115–1.129)
Unknown	113	96	1.049 (0.701–1.570)	0.569 (0.325–1.093)
Caregiver on ART				
No	47	38	1.448 (0.902–2.321)	1.485 (0.850–2.595)
Yes	41	48	1	1

n = sample included during data analysis

*Unmarried* is merged from (single, separated, divorced and widowed); *Unemployed* is merged from (housewife, farmer, student)

Italic values indicate significant variables in the multivariable analysis

### Discussion

In this study, the overall proportion of late ART initiation among children on ART was 53.2% (95% CI 48.5–58.4%). It is in line with studies of Uganda 54.4% [7], Kenya 53% [18] and Addis Ababa, Ethiopia 58.3% [11]. Conversely, the finding is higher than a study in Mozambique 48% [10]. This discrepancy might be explained by large sample size and done in district hospital using follow up study in Mozambique.

However, it is lower than studies of Uganda 72% [7, 8] in that they defined late presentation for initiation as: those at stage III and IV and for those greater than 35 month of age with aCD4 count of < 350 cells/mm^3^, whereas our study used CD4 count < 250 cells/mm^3^ for those 6 years and above. Our finding is also lower than a study in South Africa 68.2% [9] which used CDC clinical stage for late ART initiation; whereas we used both CD4 count/percentage and WHO clinical staging.

Additionally, the findings our study also lower than a study in Rural Zambia 60% and 72.4% [19, 20] and Democratic Republic Congo 66.8% [21]. This could be due to the variation in sample size and study design.

In this study, the odds of being late ART initiated among children with age **<** 5 years were two times higher as compared to those children with **>** 5 years of age. This is supported by studies of Uganda and Zambia which showed that younger age was associated with late initiation of ART [7, 8, 19]. This is due to passive immunity from maternal milk prevents signs of immune suppression. Hence, caregivers may not appreciate the problem which in turn delays ART initiation.

Moreover, the odds of late ART initiation among children of rural community were nearly two times higher as compared to those urban residents. This finding was supported by studies from Uganda and systematic review conducted in developing countries, respectively [8, 22]. It is due to high transportation cost, inaccessibility of ART service and tedious long distance traveling to reach health facilities.

Those children who took medication before ART were two times more likely to initiate ART lately as compared to those who don’t. A Zambian study indicates that concurrent treatment like TB was associated with late ART initiation [19] since it causes fear of pill burden and advanced disease.

Similarly, those children who had a past opportunistic infection before or at enrollment were nearly two and half times more likely to initiate ART lately as compared to those who didn’t have it. It is supported by a study from South Africa [9] in that the principle of treating opportunistic infection first may delay initiation of ART.

The odds of late ART initiation among children from unmarried caregivers were nearly two times as compared to children from married caregiver. It is supported by a Mozambique study in that being single was significantly associated with late ART initiation [23], since married caregivers were more stable and brought their children early for HIV testing and ART initiation.

The odds of late ART initiation among children from male caregiver were two times higher as compared to those from female caregiver. Studies of Kenya and Zimbabwe [24, 25] supported this finding in that male caregiver had extra activities out of home and engaged to run out economic affairs in the household than to carryout child rearing duties. Additionally, females have a chance of access to information about early diagnosis and initiation of ART for their children during family planning service, vaccination and antenatal follow up.

The odds of late ART initiation among children born from mothers who have null ANC visit were nearly two times more likely as compared to those from fully attended mothers. It is supported with an Ethiopia study [26] in that mothers who had antenatal follow up have excellent chance of learning about mother-to-child transmission of HIV, its early diagnosis and initiation of ART for their children.

#### Implications for clinical practice and research

This finding indicates that emphasis should be given for children who are under-5 years, rural residents, take any medicine before ART, experience past opportunistic infections, and for those from unmarried caregivers, male caregivers and mothers with null ANC visits. In line with this, researchers need to development and implement a risk prediction tool for identifying children with HIV that are likely to start ART lately. Moreover, clinicians should have a high index of suspicion in children with the aforementioned significant factors.

### Conclusion

There is high burden of late ART initiation among children on ART at UoGCSH. Age of children below 5 years, rural residence, any medication before ART initiation, presence of past opportunistic infection, null ANC follow up, unmarried and male caregivers were factors associated with late ART initiation among children on ART.

## Limitation

Since we used secondary data, variables like disclosure status, income and educational status were missed due to data incompleteness. Additionally, psychosocial issues of caregiver and children haven’t been addressed with this study.

## Abbreviations

AIDS: acquired immune deficiency syndrome
ANC: antenatal care
AOR: adjusted odds ratio
ART: anti-retroviral therapy
CD4: cluster of differentiation 4
COR: crude odds ratio
HAART: highly active anti-retroviral therapy
HIV: human immunodeficiency virus
INH: isonicotinic acid hydrazide
OIs: opportunistic infections
PMTCT: prevention of mother to child transmission
TB: tuberculosis
UoGCSH: University of Gondar Comprehensive Specialized Hospital
WHO: World Health Organization
